# HPV prevalence and genetic predisposition to cervical cancer in Saudi Arabia

**DOI:** 10.1186/1750-9378-8-15

**Published:** 2013-05-04

**Authors:** Ghazi Alsbeih, Najla Al-Harbi, Medhat El-Sebaie, Ismail Al-Badawi

**Affiliations:** 1 Biomedical Physics, King Faisal Specialist Hospital & Research Centre, Riyadh, Saudi Arabia; 2 Radiation Oncology, King Faisal Specialist Hospital & Research Centre, Riyadh, Saudi Arabia; 3 Obstetrics & Gynecology, King Faisal Specialist Hospital & Research Centre, Riyadh, Saudi Arabia; 4 Research Centre, Biomedical Physics Department, KFSHRC, MBC-03, P.O. Box 3354, Riyadh 11211, Saudi Arabia

**Keywords:** Cervical cancer, Human papillomavirus (HPV), Predisposition, Single nucleotide polymorphism (SNP)

## Abstract

**Background:**

Cervical cancer incidence is low in Saudi Arabian women, suggesting low prevalence to HPV infection due to environmental, cultural and genetic differences. Therefore, we investigated HPV prevalence and genotype distribution in cervical cancer as well as the association with 9 genetic single nucleotide polymorphisms (SNPs): *CDKN1A (p21)* C31A, *TP53* C72G, *ATM* G1853A, *HDM2* promoter T309G, *HDM2* A110G, *LIG4* A591G, *XRCC1* G399A, *XRCC3* C241T and *TGFβ1* T10C, presumed to predispose to cancer.

**Methods:**

One hundred cervical cancer patients (90 squamous cell carcinoma and 10 adenocarcinoma) and 100 age/sex-matched controls were enrolled. SNPs were genotyped by direct sequencing and HPV was detected and typed in tumors using the HPV Linear Array Test.

**Results:**

Eighty-two cases (82%) were positive for HPV sequences. Seven HPV genotypes were present as single infections (16, 18, 31, 45, 56, 59, 73) and five double infections (16/18, 16/39, 16/70, 35/52, 45/59) were detected. Most common genotypes were HPV-16 (71%), 31 (7%), and 18, 45, 73 (4% each). Only *XRCC1* SNP was significantly associated with cervical cancer (*P*=0.02, OD=1.69; 95% CI= 1.06–2.66). However, nested analysis revealed a preponderance of HPV-positivity in patients harboring the presumed risk allele *TP53* G (*P*=0.06). Both *XRCC1* and *TP53* SNPs tended to deviate from Hardy-Weinberg equilibrium (HWE; *P*=0.03-0.07).

**Conclusions:**

HPV prevalence (82%) in cervical cancer is at the lower range of the worldwide estimation (85 - 99%). While *XRCC1* G399A was significantly associated with cervical cancer, *TP53* G72C showed borderline association only in HPV-positive patients. Deviation from HWE in HPV-positive patients indicates co-selection, hence implicating the combination of HPV and SNPs in cancer predisposition. Thus, SNPs could be more relevant biomarkers of susceptibility to cervical cancer when associated with HPV infection.

## Background

Cancer of the uterine cervix is the 3rd most frequent malignancy affecting women worldwide and the seventh overall, with an estimated 530,000 new cases in 2008 [1,2]. Among all the known risk factors, human papillomavirus (HPV) stands as a main cause, and high-risk HPV infections play a major role in the pathogenesis of cervical cancer with an estimated prevalence between 85% to 99% [3-7]. More than 85% of the global burden occurs in developing countries, where it accounts for 13% of all female cancers. This is due to the lack of proper screening program that has helped reducing cervical cancer incidence and mortality rates by 70% in developed countries [8,9].

In contrast to the global view, the incidence of cervical cancer is very low in Saudi Arabia, ranking number 11 between all cancers in females and accounts only for 2.4% of all new cases [10], despite the lack of national screening programs. The actual reason for this low incidence is unknown. The closed society and standards of mores could reduce women exposure to HPV infection [11-14]. In addition, male circumcision is associated with a reduced risk of penile HPV infection and a reduced risk of cervical cancer in their female partners [15]. The prevalence of HPV infection among women and its association with cervical cancer in Saudi Arabia and in similar socio-cultural societies is scanty [14,16-20]. In a limited study performed on 120 women attending routine gynecological examination, Al-Muammar *et al.* reported a prevalence of 31.6% infection with HPV-16/18 [21]. In addition, early reports are discordant [22,23], particularly that some show high incidence, such as in Indonesia, where cervical cancer ranks number 3 after breast and colorectal tumors [2].

**Figure 1:**
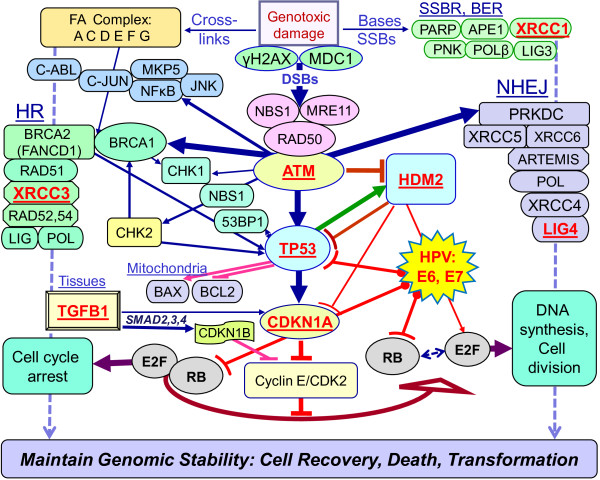
**Schematic representation of main pathways involved in processing of genotoxic DNA damage including base damages (BDs), DNA single-strand breaks (SSBs) and double-strand breaks (DSBs).** BDs and SSBs are efficiently repaired by base-excision (BER) and SSBR mechanisms. DSBs are repaired by non-homologous end joining (NHEJ) and homologous recombination (HR). These activate panoply of interacting proteins in tissues, cells and mitochondria that lead to the expression and inhibition of multiple genes. These normally results in cell cycle arrest to allow for accurate DNA healing to prevent the cells from entering DNA synthesis with damaged DNA. The aim is to maintain genomic integrity which enables recovery or otherwise triggers cell death. The E6 and E7 oncoproteins produced by high risk HPV infections will respectively interact with TP53 and RB tumor suppressor proteins and inhibit their functions leading to genomic instability. Lines represent interactions. Arrows indicate activation and blunt ends indicate inhibition. Thickness represents the strength of the actions. Underlined text designates encoding genes selected for polymorphic variations predisposing to cervical cancer (See text for details).

Inherited genetic predisposition may contribute to the risk of cervical cancer. Genetic polymorphisms in tumor suppressor genes might be related to HPV persistence and progression to cancer. The gene encoding the tumor suppressor *TP53* is one example of a candidate gene that has been suggested to affect the oncogenic potential of the HPV E6 protein. A common polymorphism in the p53 amino acid sequence is the arginine or proline at position 72 (G/C) (rs1042522). Storey *et al.* found an association between the majority allele, arginine (G) form of p53, and cervical cancer development and proposed that this genotype is more susceptible to HPV E6-mediated degradation [24]. Since then, there have been many reports on this *TP53* polymorphism and risk for cervical cancer and the results are largely contradictory [25,26]. The frequency of *TP53* codon 72 polymorphism and its relationship with HPV infection and cervical cancer is still unknown among Saudi women. In addition, TP53 is a central node in cell cycle control and DNA repair and orchestrates multiple pathways to maintain genomic integrity that can be compromised by HPV infection (Figure 1). The following SNPs: *CDKN1A* C31A Ser/Arg (rs1801270), *ATM* G1853A Asp/Asn (rs1801516), *HDM2* T309G promoter (rs2279744), *HDM2* A110G Ile/Val (rs11177386), *LIG4* A591G Ile/Val (rs2232641), *XRCC1* G399A Arg/Gln (rs25487), *XRCC3* C241T Thr/Met (rs861539) and *TGFβ1* T10C Lue/Pro (rs1982073) selected from various pathways could also alter protein function and contribute to p53-mediated cell cycle deregulation and genomic instability [27-29]. Therefore, the aims of this study were to investigate HPV prevalence and genotype in our cervical cancer patients and the potential association with these 9 genetic SNPs presumed to predispose to cancer.

## Methods

### Study population

One hundred patients with histopathologically proven, locally advanced, cervical cancer were enrolled in this study out of 218 patients followed at King Faisal Specialist Hospital and Research Centre (KFSHRC) from 2009 to 2012. There was no restriction on patients’ age or histological type of cervix cancer (squamous cell carcinoma, adenocarcinoma or other). The cervix tumor samples were obtained during routine procedure for regular biopsy or from paraffin embedded tissues. One hundred age-matched women without history of cancer were enrolled and served as normal controls. Upon signing an institutionally approved informed consent, 5-ml blood samples were withdrawn for the genetic study. The KFSHRC Research Ethics Committee has approved the study (RAC # 2060 029).

### DNA extraction, PCR amplification, DNA sequencing and SNP genotyping

DNA was extracted using Puregene DNA Purification Kit (Gentra System). The PCR primers used for amplification were published previously [28]. Relevant segments of DNA were amplified by thermal cycling (95°C for 15 min, 39 rounds of 95°C for 1 min, 56°C for 1 min and 72°C for 1 min and final extension at 72°C for 7 min) using HotStarTaq DNA polymerase (Qiagen), and 50 ng template DNA in 25 micro-litter volume with standard reaction conditions. The amplified fragment was directly sequenced using the DYEnamic ET Dye Terminator Cycle Sequencing Kit (Amersham Biosciences) and were run on the MegaBase 1000 sequencer (Applied Biosystems). Sequencing results were aligned to the corresponding reference sequence and the SNPs were genotyped using SeqManII sequence analysis software (DNASTAR Inc.).

### HPV detection and genotyping

We used the Linear Array HPV Genotyping Test **(**LA HPV GT; Roche Diagnostics). The LA HPV GT is based on four major processes including DNA extraction, PCR amplification of target DNA, hybridization of amplified products to oligonucleotide probes and finally, the colorimetric determination using the Linear Array Detection Kit (LA DK). It enables the concurrent detection and genotyping of 37 most common anogenital HPV DNA genotypes [6, 11, 16, 18, 26, 31, 33, 35, 39, 40, 42, 45, 51, 52, 53, 54, 55, 56, 58, 59, 61, 62, 64, 66, 67, 68, 69, 70, 71, 72, 73 (MM9), 81, 82 (MM4), 83 (MM7), 84 (MM8), 89 (CP6108) and IS39]. The test has the betaglobin gene as an internal control to show adequacy of the sample. The manufacturer states a sensitivity of 96% (95% CI: 92-98%) and a specificity of 99% (95% CI: 98-100%) and has included 3 controls, 2 negatives and 1 positive for HPV-16. The kit also enables detection of multiple infections. The primers and PCR reaction conditions were provided with the test kit. The manufacturer’s recommended methodology was strictly followed as also described previously [17]. Positive reactions appear as blue bands on the test strip. The strips were interpreted using the HPV reference guide provided with the kit. Results were considered negative if no HPV band was detected after at least two repeated testing.

### Statistical analysis and ethical considerations

A total of 100 patients and 100 controls subjects were included in the project following signing an informed consent. Samples were coded with no identifiable personal data. HPV status was compared between the patients in 5-year age groups. The association between SNPs and cervical cancer was evaluated by the odd ratio (OR) with its confidence interval. The degree of significance was calculated using the Chi-Squares method. A p-value of 0.05 or less was considered statistically significant. Statistical analysis was carried out using the SigmaPlot platform (Version 12.0, SPSS Science, IL, USA) and the free online software: Case Control Studies, Tests for Association, Institute of Human Genetics, Helmholtz Center Munich, Germany (http://ihg.gsf.de/cgi-bin/hw/hwa1.pl).

## Results and discussion

### Subjects and clinical data

**Figure 2:**
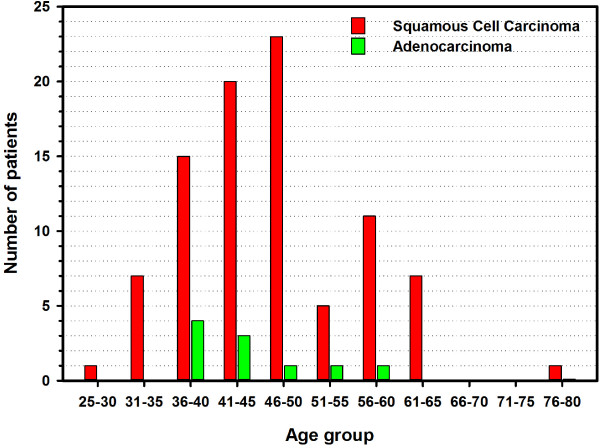
The distribution of squamous cell carcinoma and adenocarcinoma by 5-year age group in 100 cervical cancer patients.

Normal controls had similar socio-economic and demographic distribution and age range (30 to 73, median = 48) as the patients. Age of patients at diagnosis of cervical cancer ranged between 30 and 76 years, with a median of 46 years. The FIGO stage of the cancer ranged between IA2 and IVA but most patients (*n*=76) had stage II/III disease. By histology, 90 patients had squamous cell carcinoma while 10 had adenocarcinoma of the cervix. The distribution of these two histopathological types by 5-year age groups is given in Figure 2. Taking into consideration two previously published data [17,18], adenocarcinoma forms only 12% of cervical cancers compared to 88% of squamous cell carcinoma.

### HPV detection and genotyping

HPV detection and genotyping showed that 82 patients (82%) were positive for HPV infection while 18 specimens proved to be negative (18%) after at least two independent testing. This prevalence of HPV infection in Saudi cervical cancer patients is at the lower range of the estimated 85% to 99% worldwide [5,7,30], and also contrasting with the high burden estimated from previously published data from the extended Middle East and North Africa that showed up to 98% positivity in women with preinvasive and invasive lesions [31]. Khorasanizadeh *et al.* has reported slightly lower prevalence (76%) in a nearby country [19].

**Figure 3:**
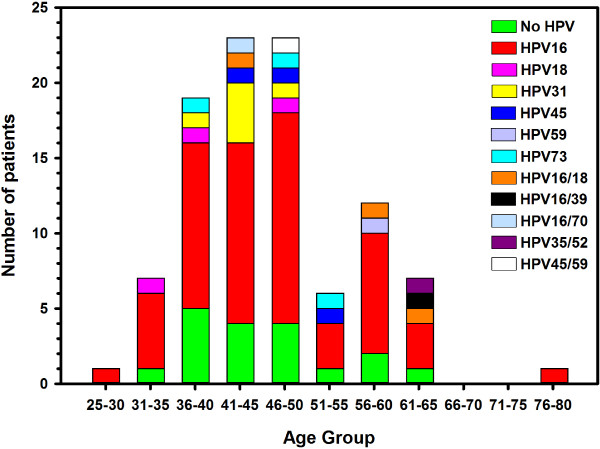
The distribution of HPV detection and genotypes by 5-year age group in 100 cervical cancer patients.

By histology, 60% of adenocarcinomas and 84% of squamous cell carcinomas were HPV-positive. Linear Array HPV genotyping test had detected seven different single HPV genotypes and five double infections in this cohort. Results are summarized in Table 1. The age distribution of HPV detection and genotypes is shown in Figure 3. Furthermore, age-specific HPV distribution in the Saudi cervical cancer patients showed a bimodal curve with a first peak at younger ages (41–50 years) and a relative rebound at older ages (56–60 years) as it has been described in other population [32].

**Table 1:** Detection and Prevalence of different HPV genotypes in 100 cervical cancer patients

**HPV genotypes**	**Classification**	**Number of patients**	**Prevalence (%)**
HPV-positive		82	82%
HPV-negative		18	18%
**Single infection:**			
HPV-16	HR	58	70.73
HPV-18	HR	3	3.66
HPV-31	HR	6	7.32
HPV-45	HR	3	3.66
HPV-56	HR	1	1.22
HPV-59	HR	1	1.22
HPV-73	HR	3	3.66
**Co-infections:**			
HPV-16/18	HR/HR	3	3.66
HPV-16/39	HR/HR	1	1.22
HPV-16/70	HR/LR	1	1.22
HPV-35/52	HR/HR	1	1.22
HPV-45/59	HR/HR	1	1.22

LR: low risk; HR: high risk.

The most common HPV genotypes were HPV-16 (71%), followed by HPV-31 (7%), HPV-18, 45, 73 (4% each). Seven patients had double infections involving HPV-16/18 (4%), HPV-16/39, 16/70, 35/52, and 45/59 (1% each). In agreement with other studies, the most common HPV genotype was HPV-16 [33] with an overall prevalence, including co-infections, of 77% compared to 54% in the world [30]. In addition, HPV-16 and/or HPV-18 were present in 66% (66/100) of all patients and formed together 80% (66/82) of all HPV positive patients. This is slightly higher than the prevalence observed in Europe (74.5%), North America (76.5%) and in the whole world (70.9%). These results are close to those reported in another neighboring country where HPV-16 (54%), HPV-18 (14%), and HPV-31 (6%) were the most commonly detected in cervical cancer patients [19]. However, our results seem to be different from those obtained in other Middle Eastern country where the most common HPV genotype was HPV-33, which was not detected in our patients, followed by HPV-16 and HPV-18 [34].

### Genetic polymorphic variations

Genetic polymorphic variation in candidate SNPs were determined for all 100 cervical cancer patients in addition to the 100 age-matched female volunteers without cancer (controls). The two SNPs, *LIG4* rs2232641 and *HDM2* rs11177386 were all majority alleles, and therefore, were omitted from the analysis. Cancer predisposition study showed significant association between *XRCC1* rs25487 and having cervical cancer (Table 2). Patients harboring the variant allele (*A*, Gln) have about 2-fold increased risk to develop cervical cancer (*P* = 0.02). These results suggest that the variant (or minority) allele could confer higher susceptibility to cervical cancer and probably the HPV-related transformation.

**Table 2:** Genotypes’ and alleles’ frequencies of 7 assessed polymorphisms in 100 cervix carcinoma patients in addition to 100 age-and-gender matched control volunteers without cancers

**Gene, genotype and allele**	**Cancer patients’ frequency**	**Normal volunteers’ frequency**	**Odds ratio**	***P*- Value**
			**(95% CI)**	
	**(%, *n *= 100)**	**(%, *n *= 100)**		
*CDKN1A (p21)* codon 31 C>A Ser/Arg rs1801270				
C/C	64 (64)	62 (62)		
C/A	32 (32)	35 (35)	0.89 (0.49-1.60)	0.69
A/A	4 (4)	3 (3)	1.29 (0.28-6.01)	0.74
C	160 (80)	159 (79)		
A	40 (20)	41 (21)	0.97 (0.59-1.58)	0.90
*TP53 (p53)* codon 72 G>C Arg/Pro rs1042522				
G/G	20 (20)	22 (22)		
G/C	58 (58)	52 (52)	1.23 (0.60-2.50)	0.57
C/C	22 (22)	26 (26)	0.93 (0.41-2.14)	0.87
G	98 (49)	96 (48)		
C	102 (51)	104 (52)	0.96 (0.65-1.42)	0.84
*ATM* codon 1853 G>A Asp/Asn rs1801516				
G/G	90 (90)	88 (88)		
G/A	8 (8)	12 (12)	0.65 (0.25-1.67)	0.37
A/A	2 (2)	0 (0)	4.89 (0.23-103.2)	0.16
G	188 (94)	188 (94)		
A	12 (6)	12 (6)	1.00 (0.44-2.28)	1
*HDM2* promoter 309 T>C rs2279744				
T/T	30 (30)	27 (27)		
T/C	46 (46)	44 (44)	0.94 (0.48-1.83)	0.86
C/C	24 (24)	29 (29)	0.75 (0.35-1.58)	0.44
T	106 (53)	98 (49)		
C	94 (47)	102 (51)	0.85 (0.58-126)	0.42
*TGFβ1* codon 10 T>C Leu/Pro rs1982073				
T/T	48 (48)	39 (39)		
T/C	31 (31)	38 (38)	0.89 (0.42-1.91)	0.77
C/C	21 (21)	23 (23)	1.35 (0.65-2.79)	0.42
T	127 (64)	116 (58)		
C	73 (37)	84 (42)	1.26 (0.84-1.88)	0.26
*XRCC1* codon 399 G>A Arg/Gln rs25487				
G/G	52 (52)	59 (59)		
G/A	34 (34)	40 (40)	0.96 (0.54-1.74)	0.90
A/A	14 (14)	1 (1)	15.88 (2.0-124.9))	0.0007
G	138 (69)	158 (79)		
A	62 (31)	42 (21)	1.69 (1.07-2.66)	0.02
*XRCC3* codon 241 C>T Thr/Met rs861539				
C/C	45 (45)	41 (41)		
C/T	44 (44)	37 (37)	1.08 (0.59-1.99)	0.79
T/T	11 (11)	22 (22)	0.46 (0.20-1.05)	0.06
C	134 (67)	119 (60)		
T	66 (33)	81 (41)	0.72 (0.48-1.09)	0.12

Indeed, the analysis of this nested case–control study shows that 93% (13/14) of patients with homozygous variant alleles (*A/A*) are HPV-positive compared to 82% (27/33) in heterozygous and 79% (42/53) in majority allele (*G/G*), suggesting a trend toward an association between the HPV-positivity and *XRCC1* G399A genotype; however, it did not reach statistical significance (*P* = 0.28; Table 3). To check for skewness in the distribution of *XRCC1* G399A genotypes, we tested for deviation from Hardy-Weinberg equilibrium (HWE), by comparing observed-to-expected distributions in HPV-positive (cases) and HPV-negative (controls) patients (Table 3). Results showed statistically significant deviation from HWE for cases (*P* = 0.03) but not for the controls. Therefore, in HPV+ cervical cancer patients, the null hypothesis that the population is in Hardy–Weinberg frequencies is rejected, which put forward the hypothesis of probable selection. This suggests that the co-occurrence of *XRCC1* G399A genotypes and HPV-positive cancer is not random, thus, implicating this SNP as a susceptibility locus to develop cervical cancer. At the molecular level, *XRCC1* protein is required for efficient DNA single-strand breaks repair to maintain genomic stability in human cells (Figure 1) and its reduction leads to increased sensitivity to cell killing by ionizing radiation [35]. The codon 399 is situated in the BRCT I active domain of the protein and could possibly affect its function [36]. In addition, this SNP was associated with cellular and clinical sensitivity to cancer treatment [37] and has recently been implicated in susceptibility to cervical cancer among Asian women [38].

**Table 3:** Genotypes’ association with HPV status and test for deviation from Hardy-Weinberg Equilibrium

**Gene, genotype and allele**	**HPV+/− *n *( *% *)**		**Odds ratio**	***P*-Value**	***Deviation from HWE***	
	**Cases**	**Controls**	**(95% CI)**		**Expected**	
	**(HPV+) (*n *= 82)**	**(HPV-) (*n *= 18)**			**Cases**	**Controls**
*XRCC1* codon 399 G>A Arg/Gln (rs25487)						
*Genotype*						
G/G	42 (51)	11 (61)			37.56	10.89
G/A	27 (33)	6 (33)	1.18 (0.39-3.56)	0.77	35.87	6.22
A/A	13 (16)	1 (6)	3.41 (0.40-28.93)	0.24	8.56	0.89
G/A+A/A	40 (49)	7 (39)	1.49 (0.53-4.24)	0.45		
*Allele*						
G	111 (68)	28 (78)				
A	53 (32)	8 (22)	1.67 (0.71-3.91)	0.23		
Armitages’ trend test			Common OR= 1.67	0.28		
Significance level (*P*-value) for deviation from HWE					0.03	0.88
*TP53 (p53)* codon 72 G>C Arg/Pro (rs1042522)						
*Genotype*						
G/G	18 (22)	2 (11)			22.03	2.35
G/C	49 (60)	9 (50)	0.61 (0.12-3.07)	0.54	40.95	8.31
C/C	15 (18)	7 (39)	0.24 (0.04-1.32)	0.08	19.03	7.35
G/C+C/C	64 (78)	9 (89)	0.44 (0.09-2.12)	0.29		
*Allele*						
G	85 (52)	13 (36)				
C	79 (48)	23 (64)	0.53 (0.25-1.11)	0.09		
Armitages’ trend test:			Common OR= 0.48	0.06		
Significance level (*P*-value) for deviation from HWE					0.07	0.72

HWE: Hardy-Weinberg Equilibrium. OR: Odds Ratio.

Computed online at: http://ihg.gsf.de/cgi-bin/hw/hwa1.pl.

In contrast, no association was found for *TP53* G72C where cancer patients and controls without cancer have showed similar frequencies (Table 3). Nonetheless, nested analysis showed that 90% of patients with majority (G/G) allele were HPV-positive compared to 74% of heterozygous (G/C), and 68% of homozygous (C/C) variant allele, revealing a preponderance of HPV-positivity in patients harboring the majority (G) allele. In fact, this allele has been suggested to be more susceptible to high-risk HPV E6 degradation [24]. In addition, statistical analysis showed a trend towards an association between *TP53* G72C SNP genotype and HPV infection (*P* = 0.06; Table 3). Furthermore, testing for deviation from HWE also showed a borderline significant deviation (*P* = 0.07) for HPV-positive cases, meanwhile no significant deviation was observed for HPV-negative controls. Again, these results suggest that cervical cancer occurrence is not random in the population and that certain factors such as genetic SNPs, for instance having the *XRCC1* A-allele or the *TP53* G-allele in connection with HPV infection, favors its development. To answer the question whether these 2 SNPs exhibit cumulative effect towards HPV mediated cervical cancer, , we have computed the number of risk alleles for *XRCC1* and *TP53* in patients and controls. Although the patients had higher median number of risk alleles (2 compared to 1), the difference was not statistically significant (Mann–Whitney rank sum test, *P* = 0.12) suggesting independent effects.

Since the first identification of the potential role of Arg/Arg genotype as a risk marker for uterine cervix neoplasia [24], there have been many studies that investigated the association between the *TP53* codon 72 polymorphism and cervical cancer in various populations; however, results were inconsistent [29]. Although several factors were proposed as contributing factors to the discrepancies, the deviation from the Hardy-Weinberg equilibrium was identified as a principal source of divergent results [39]. Nonetheless, there is sufficient evidence to support a positive association particularly when HPV status and/or histopathology is known. Thus, while Sousa et al. failed to confirm the association in most European countries, except *Italy* and *United Kingdom*[40], two other meta-analyses confirmed the association of homozygous Arg with invasive cervical cancer [39,41], In line with our results, a recent family-based association study where HPVs status was also determined, Hu *et al.* confirmed that the *TP53* codon 72 G (arginine) is significantly overtransmitted in Caucasian cervical cancer subjects, especially in cases infected with HPV16- and/or HPV-18 [29].

## Conclusions

The prevalence of HPV infection in invasive cervical cancer in Saudi Arabia (82%) is at the lower range of that observed in the world (85%-99%), the most common HPV genotype was by far HPV-16 (71%), followed by HPV-31 (7%), HPV-18, 45, and 73 (4% each) with double infections were present in 8.5% of HPV-positive patients. Genetic predisposition showed that among the nine SNPs studied, only *XRCC1* G399A was significantly (*P* = 0.02) associated with cervical cancer, while *TP53* G72C showed borderline association (*P* = 0.06) only in HPV-positive patients. In addition, both SNPs showed degrees of deviation from Hardy-Weinberg equilibrium only in HPV-infected tumors, indicative of non-random distribution, hence implicating the combination of HPV and SNPs in cancer predisposition. Thus, SNPs could be more relevant biomarkers of susceptibility to cervical cancer when associated with HPV infection. Further studies with larger cohort are needed to confirm these results and better postulate the use of SNPs as biomarkers of susceptibility to cervical cancer.

## Abbreviations

SNP: Single nucleotide polymorphism; HPV: Human papilloma virus; CDKN1A: *c*yclin-dependent kinase inhibitor 1A; TP53: Tumor protein p53; ATM: Ataxia-telangiectasia mutated; HDM2: Human double minutes 2 (also known as MDM2); LIG4: DNA ligase IV; XRCC1: X-ray repair cross-complementing 1; XRCC3: X-ray repair cross-complementing 3; TGFB1: Transforming Growth Factor Beta 1; HWE’: Hardy-Weinberg equilibrium; OD: Odd Ratio; CI: Confidence interval.

## Competing interests

The authors declare that there are no conflicts of interest related to this research manuscript.

## Authors’ contributions

GA designed the study, analyzed results and drafted manuscript. NAH processed and genotyped samples. MES and IA selected study population, gynecological sampling and patient care. All authors read and approved the final manuscript.
